# Multi-strategy ugt mining, modification and glycosyl donor synthesis facilitate the production of triterpenoid saponins

**DOI:** 10.3389/fpls.2025.1586295

**Published:** 2025-05-30

**Authors:** Lin Hao, Yu Liu, Guiru Dong, Jingyan Liu, Kai Qiu, Xiaopeng Li, Yanan Qiao

**Affiliations:** ^1^ School of Pharmacy, Shandong Second Medical University, Weifang, Shandong, China; ^2^ Chemical Drug Testing Laboratory, Weifang Inspection and Testing Center, Weifang, Shandong, China; ^3^ Dongying High level Talent Research Center, Dongying, Shandong, China

**Keywords:** triterpenoid saponins, glycosylation, UDP-glycosyltransferases (UGTs), multi-omics approaches, enzyme engineering

## Abstract

Triterpenoid saponins are a class of plant secondary metabolites with significant biological activities and are widely used in the pharmaceutical and nutritional supplement industries. However, the production of triterpenoid saponins is limited by their complicated biosynthetic pathways and the availability of glycosyl donors. UDP-glycosyltransferases (UGTs) play a key role in the glycosylation of triterpenoid saponins, significantly enhancing their structural diversity, solubility, pharmacological activity, and bioavailability. Therefore, the identification and modification of efficient, specific, and stable UGTs have attracted attention. This review focused on the advances in the glycosylation of triterpenoid saponins, with a particular emphasis on the application of multi-omics approaches in UGT mining. The combination of genomics, transcriptomics, and metabolomics has provided powerful tools for UGT screening, significantly improving the efficiency and accuracy of UGT identification. Additionally, the methods based on gene clusters, phylogenetic analysis, and the plant secondary product glycosyltransferase (PSPG) motif also offer new perspectives for UGT identification. Besides, the application of synthetic biology platforms has provided innovative approaches for high-throughput screening and functional validation of UGTs, laying a theoretical foundation for the functional modification of UGTs. We also discussed the latest research progress on UGT modification including directed evolution and rational design. These strategies, through amino acid mutations and structural optimization, are expected to enhance UGT catalytic activity, thermal stability, and broaden substrate specificity. Moreover, the diversity and availability of glycosyl donors directly influence the efficiency of glycosylation reactions and the diversity of the products. Thus, we discussed glycosyl donor synthesis, including *in vitro* and *in vivo* synthetic strategies. By optimizing metabolic pathways and introducing key enzyme genes, engineered microorganisms can efficiently synthesize various glycosyl donors, providing abundant substrates for glycosylation reactions. These studies offer new opportunities and challenges for the synthesis and application of triterpenoid saponins, promoting their industrial potential.

## 1 Introduction

**Figure 1:**
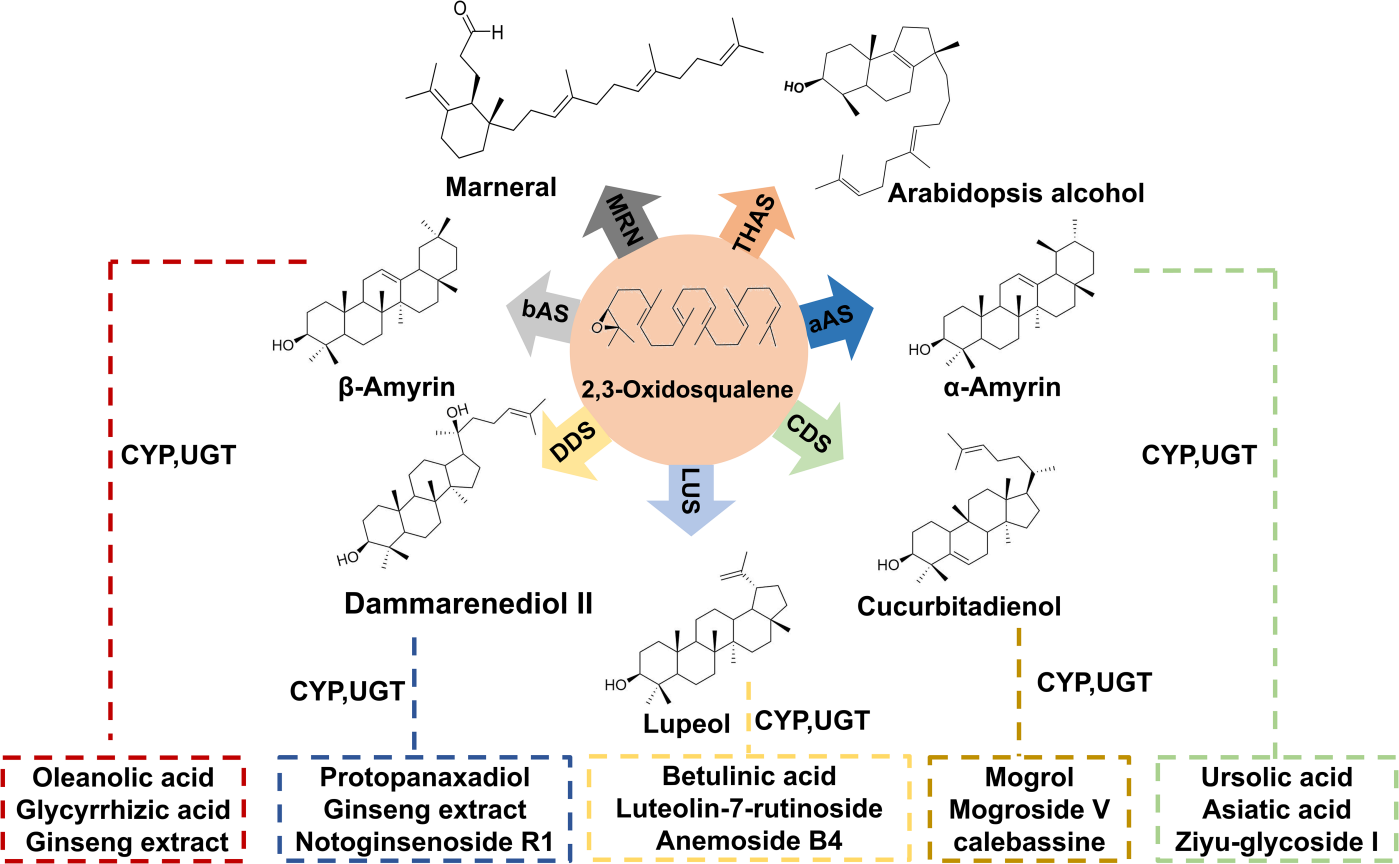
Classification diagram of partial triterpenes.

**Figure 2:**
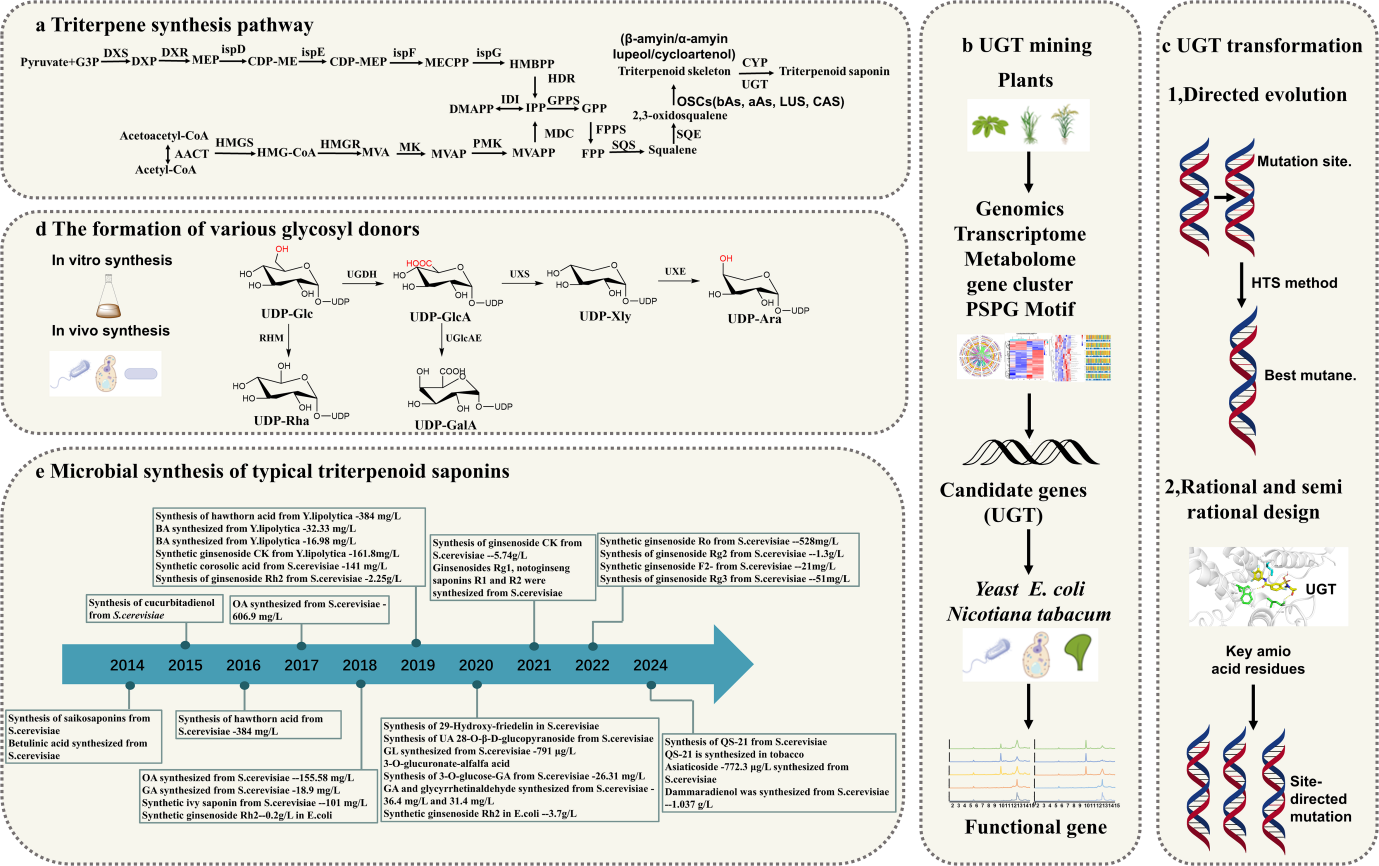
Overview of Triterpene Glycosyltransferase Research. **(a)** Triterpene Biosynthetic Pathways; **(b)** Discovery of UGTs (Uridine 5’-diphosphate Glucosyltransferases); **(c)** Modification of UGTs; **(d)** Glycosyl donor Biosynthetic Pathways; **(e)** Microbial Production of Triterpene Compounds.

Triterpenoids are a class of terpenoid compounds with a basic skeleton formed by six isoprene units (C_5_H_8_). They can exist in free form or be conjugated with sugars to form glycosides or esters. Notably, triterpenoid saponins are widely distributed in nature and demonstrate significant pharmacological activities, including anti-inflammatory, antifungal, antibacterial, antiparasitic, and anticancer effects (Singh et al., 2024; Xu et al., 2024; Dong et al., 2023) (**Figure 1**). The biosynthesis of triterpenoids begins with the condensation of isopentenyl pyrophosphate (IPP) and dimethylallyl pyrophosphate (DMAPP), catalyzed by farnesyl pyrophosphate synthase (FPPS) to form the C_15_ molecule farnesyl pyrophosphate (FPP). Two molecules of FPP undergo a “head-to-head” condensation to form the linear C_30_ molecule squalene, which is then oxidized to yield the important precursor 2,3-oxidosqualene, possessing the triterpene skeleton. Squalene is oxidized to 2,3-oxidosqualene by the squalene epoxidase (SQLE) enzyme, which is a cytochrome P450 enzyme. This oxidation step is crucial as it introduces an epoxy group at the 2,3 positions of squalene, forming a reaction intermediate that serves as a branch point for different triterpene biosynthetic pathways. SQLE uses molecular oxygen and NADPH as cofactors to facilitate this oxidation process, which is the rate-limiting step in triterpene biosynthesis. 2,3-oxidosqualene enters various branched metabolic pathways under the action of different oxidosqualene cyclases (OSCs), leading to the formation of various cyclic precursors (Hazra et al., 2023) (**Figure 2a**). Next, the structural diversity of triterpenes arises from cyclization and oxidation steps, their pharmacological versatility is further amplified by glycosylation—a process mediated by UGTs (Li et al., 2023; Hucheng et al., 2023), which transfer activated sugar units from sugar nucleotide donors to a receptor, forming stable glycosidic bonds. According to the CAZy database, enzymes responsible for the glycosylation of small, lipophilic molecules in plants belong to the Glycoside Hydrolase Family 1 (GT1) and UDP-dependent glycosyltransferase (UGT) superfamily. Although more than 20,000 members have been identified in this family, less than 2% of them have been characterized (CAZy database statistics). UGTs play a key role in the structure and pharmacological properties of triterpenoid saponins (Gloster, 2014; Wilson and Tian, 2019).

Multi-omics methods including genomics, transcriptomics, and metabolomics, have become powerful tools mining UGT genes involved in triterpenoid saponins biosynthesis, enabling the systematic correlation of gene expression, metabolite profiles, and enzyme activity. Besides, genome clustering, phylogenetic analysis, and PSPG motif-based screening methods have offered new perspectives for identifying UGTs (Wang et al., 2023a). The PSPG motif is a conserved domain involved in glycosyl donor binding and is particularly important for identifying functional UGTs (Andong et al., 2022). Furthermore, the application of synthetic biology platforms has provided innovative methods for the high-throughput mining and functional validation of UGTs.

Glycosylation efficiency fundamentally depends on glycosyl donor availability. The diversity and accessibility of these donors directly influence reaction efficiency and product diversity. Key donor molecules include D-glucose (D-Glu), D-galactose (D-Gal), D-glucuronic acid (D-GlcA), L-rhamnose (L-Rha), D-xylose (D-Xyl), and L-arabinose (L-Ara), as established in current research (Kurze et al., 2022). This highlights the essential role of UDP-sugar biosynthesis in glycoside production. Existing manufacturing strategies divide into chemical synthesis and biological approaches. Due to the structure complexity of glycosyl donors, the steps for their chemical synthesis are costly and highly toxic, making it difficult to achieve their mass production., while engineered microbial systems (e.g., *E. coli*, yeast) have become main production platforms. Through metabolic engineering and enzyme optimization, these biological systems have demonstrated potential donor synthesis capabilities, overcoming traditional limitation of substrates (Cravens et al., 2019; Lu et al., 2023).

Recent studies have prioritized pathway engineering and heterologous production over basic UGT characterization, emphasizing integrated multi-omics and synthetic biology frameworks (Reed et al., 2023; Zhao et al., 2024; Xu et al., 2024). This review systematically examined advancements in triterpenoid glycosylation research, particularly highlighting multi-omics strategies for UGT discovery (Li et al., 2023; Wang et al., 2021a). We investigated innovative engineering tactics spanning genetic/protein modifications and evaluates both chemical and biological glycosyl donor production methods. These developments create new possibilities for optimizing triterpenoid saponin biosynthesis while presenting challenges for industrial applications (**Figure 2**).

## 2 Multi-strategy UGT mining for triterpene biosynthesis

UGTs enzymes play critical roles in the post-modification of triterpenoid compounds, significantly contributing to their structural diversity. However, many UGT genes responsible for these modifications remain largely unexplored. Extensive studies have been performed on glucosyltransferases and glucuronic acid transferases, while far fewer investigations have focused on other UGTs, such as those responsible for the transfer of galactose, xylose, mannose, fucose, and arabinose (Li et al., 2014). In recent years, the omics technologies have provided new perspectives and tools for the identification and characterization of UGTs involved in triterpene glycosylation (**Table 1**). The combination of genomics, transcriptomics, and metabolomics allows researchers to systematically analyze the functions and mechanisms of UGTs from multiple dimensions (Dai and Shen, 2022). Additionally, strategies based on gene clusters, phylogenetic analysis and the presence of PSPG motifs have enhanced the efficiency and accuracy of UGT mining (**Figure 2b**). Furthermore, the application of synthetic biology platforms offers innovative approaches to explore and engineer these enzymes for improved catalytic efficiency and specificity. This review comprehensively explored the application of multi-omics approaches in the screening of UGTs related to triterpene glycosylation. It also analyzed strategies based on gene clusters, phylogenetic analysis and PSPG motifs, and discuss the advancements in synthetic biology platforms.

**Table 1:** Identification method for glycosyltransferase of triterpenoids.

Gene	source	Function	Gene ID	Excavation means	References
UGT73CZ2	*Q. saponaria*	glycosyl transferase	OQ241425.1	Transcriptome,Genome, Gene cluster	(Martin et al., 2024)
CSLM1, CSLM2	*Q. saponaria*	UDP-glucuronyl transferase	OQ107253.1 OQ107265.1	Transcriptome,Genome, Gene cluster	(Liu et al., 2024)
UGT73CU3	*Q. saponaria*	UDP-galactosyl transferase	OQ107259.1	Transcriptome,Genome, Gene cluster	(Liu et al., 2024)
UGT73CX1	*Q. saponaria*	C3-xylosyltransferase	OQ107254.1	Transcriptome,Genome, Gene cluster	(Liu et al., 2024)
UGT73CX2	*Q. saponaria*	C3-rhamnosyl transferase	OQ107255.1	Transcriptome,Genome, Gene cluster	(Liu et al., 2024)
UGT74BX1	*Q. saponaria*	C28-fucosyltransferase	OQ107250.1	Transcriptome,Genome, Gene cluster	(Liu et al., 2024)
UGT91AR1	*Q. saponaria*	C28-rhamnose transferase	OQ107251.1	Transcriptome,Genome, Gene cluster	(Liu et al., 2024)
UGT91AQ1	*Q. saponaria*	C28-xylosyltransferase	OQ107264.1	Transcriptome,Genome, Gene cluster	(Liu et al., 2024)
UGT73CY3	*Q. saponaria*	C28-xylosyltransferase	OQ107263.1	Transcriptome,Genome, Gene cluster	(Liu et al., 2024)
UGT73CY2	*Q. saponaria*	C28-apiose	OQ107262.1	Transcriptome,Genome, Gene cluster	(Liu et al., 2024)
UGT1A1	*G. uralensis*	Udp-glucuronide transferase	–	Genome	(Xu et al., 2021)
*Gu*GT14	*G. uralensis*	UDP-glucuronyl transferase	MK534521.1	Genome	(Xu et al., 2021)
*Gu*UGT73P12	*G. uralensis*	UDP-glucuronyl transferase	BBN60804	Genome, Transcriptome	(Xu et al., 2021)
*Pg*UGT71A27	*P. ginseng*	C20-glycosyltransferase	KM491309.1	Transcriptome	(Le et al., 2021)
*Pg*UGT71A53	*P. ginseng*	C20-xylosyltransferase	–	Transcriptome	(Le et al., 2021)
*Pg*UGT74AE2	*P. ginseng*	C3-glucosyltransferase	A0A0A6ZFR4	Transcriptome	(Le et al., 2021)
*Pg*UGT71A54	*P. ginseng*	C6-xylosyltransferase	–	Transcriptome	(Le et al., 2021)
*Pg*UGT94Q2	*P. ginseng*	C3/C20-glucuronyl transferase	A0A0A6ZFY4	Transcriptome	(Le et al., 2021)
*Pg*UGT74AE4	*P. ginseng*	C3-glycosyl transferase	–	Transcriptome	(Le et al., 2021)
*Pv*fUGT1, *Pv*fUGT2	*P. Vietnamensis*	C20/C24-glycosyltransferases	–	Transcriptome	(Sufang et al., 2024)
*Pq*UGT1	*P. quinquefolius*	glycosyl transferase	–	Transcriptome	(Bihuan et al., 2024)
*Ae*CSL1	*A. elata*	C3-glycosyl transferase	AE06G00237	Transcriptome	(Hyokchol, 2024)
*Ae*CSL2	*A. elata*	C3-glycosyl transferase	AE06G00238	Transcriptome	(Hyokchol, 2024)
*Pn*UGT3498	*P. notoginseng*	C3-glycosyl transferase	–	Transcriptome	(Li, 2023)
*Pn*UGT4291	*P. notoginseng*	C3-glycosyl transferase	–	Transcriptome	(Li, 2023)
*Pn*UGT6350	*P. notoginseng*	C3-glycosyl transferase	–	Transcriptome	(Li, 2023)
*Am*GT11	*A. membranaceus*	glycosyl transferase	–	Transcriptome, Genome	(Xu et al., 2024)
*Am*GT36	*A. membranaceus*	6-O-glucosylation	–	Transcriptome, Genome	(Xu et al., 2024)
*Am*UGT7	*A. membranaceus*	C3-glycosyl transferase	–	Transcriptome	(Duan et al., 2023)
*Am*UGT15	*A. membranaceus*	C6-glycosyl transferase	–	Transcriptome	(Duan et al., 2023)
*Am*GT1, *Am*GT5	*A. membranaceus*	C3-glycosyl transferase	A0A2D2CI62 -	Transcriptome	(Barbi et al., 2010)
*Am*GT9	*A. membranaceus*	C25-glycosyltransferase	–	Transcriptome	(Barbi et al., 2010)
*Ac*CSL1	*A. chinensis*	C3-glycosyl transferase	A0A0A9XC01	Metabolome,genome, transcriptome, gene cluster	(Sun et al., 2023b)
OAGT	*P. zingiberensis*	UDP-glucuronyl transferase	A0A385MJ20	Transcriptome	(Tang et al., 2019)
UGT73C10, UGT73C11	*B. vulgaris*	C3-glucuronyl transferase	AFN26666.1	CDNA expression library screening	(Augustin et al., 2012)
UGT73C33	*G. uralensis*	C3-glucuronyl transferase	–	Genome	(Zhang et al., 2020)
UGT73F24	*G. uralensis*	C30-glucuronyl transferase	–	Genome	(Zhang et al., 2020)
*Gm*CSyGT1	*G. max*	C3-glucuronyl transferase	Glyma.06G324300	Transcriptome,co-expression	(Chung et al., 2020)
*Gu*CSYGT	*G. uralensis*	C3-glucuronyl transferase	Glyur003152s00037491	Transcriptome,co-expression	(Chung et al., 2020)
*Lj*CSYGT	*L. japonicus*	C3-glucuronyl transferase	Lj3g3v1981230	Transcriptome,co-expression	(Chung et al., 2020)
*Ca*UGT1	*C. roseus*	C28-glucuronyl transferase	C6ZRH7	Transcriptome	(Kaminaga et al., 2004)
*Ca*UGT73C7 *Ca*UGT73C8	*C. asiatica*	C28-glucuronyl transferase	CM025783.1.1337 CM025783.1.1336	Transcriptome	(Zhao et al., 2024)
UGT74AG5	*I. asprella*	UDP-glucuronyl transferase	–	Transcriptome	(Ji et al., 2020)

### 2.1 Multi-omics approaches for screening glycosyltransferases involved in triterpenoid glycosylation

Plant genomics and transcriptomics have provided extensive insights into structural genes, facilitating the identification of those involved in the biosynthesis of valuable secondary metabolites in plants (Jendoubi, 2021). Among various multi-omics approaches, genomics clarifies the structure, function, evolution, localization, and editing of genomes (You, 2023). Transcriptomics focuses on identifying and quantifying RNA, offering valuable insights into gene expression profiles under specific conditions and over time (Zhang et al., 2023). Metabolomics complements genomics and transcriptomics by uncovering the metabolic responses of organisms to external stimuli, environmental changes, or genetic modifications, thus providing a comprehensive view of cellular processes (**Figure 2b**). By integrating data from these three omics, researchers can systematically identify and screen UGT candidates involved in the biosynthesis of triterpenoids (Alami et al., 2023; Song et al., 2023).

### 2.1.1 Genome-based screening of glycosyltransferases involved in triterpenoid glycosylation

In plant genomics, gene annotation technology provides important clues to parse key DNA sequences. By tracking the common features of plant genomes, researchers can more clearly delineate evolutionary trajectories and taxonomic relationships. Notably, genome annotation data can also aid in the prediction of potential UGTs candidate genes (Song et al., 2023). Taking the *G. uralensis* genome study as an example, the Xu team accurately targeted UGT1A1 from 22 candidate UGTs and confirmed that the enzyme has efficient catalytic characteristics for the biosynthesis of glycyrrhizic acid GL and GAMG (Xu et al., 2021). The next results showed that *Gu*GT14 and *Gu*UGT73P12 could synergistically catalyze the directional connection of glucose and glucuronic acid at the C-3 position of the parent nucleus to form the characteristic glycyrrhizic acid (Xu et al., 2021). Genome analysis of *A. chinensis Bunge* discovered two gene groups for triterpene production: a 350 kb cluster containing multiple biosynthetic genes including oxygenase homologs and BAHD family members (Sun et al., 2023b). While identifying the gene clusters involved in triterpene biosynthesis by using triterpene pathway synthesis genes with known functions, such as OSCs and cleavage enzymes (CYPs), it also promotes the mining and identification of UGT on their synthetic pathways. Based on *G. uralensis* genome insights, researchers successfully identified UGT73C33, which specifically modified the 3-C position of triterpene cores (Zhang et al., 2020). This evidence confirmed that systematic examination of gene clusters associated with metabolic pathways can significantly improve the accuracy of UGT identification.

#### 2.1.2 Transcriptome-based screening of glycosyltransferases involved in triterpenoid glycosylation

Compared with genomics, transcriptomics provides information into the temporal and spatial variations in gene expression. Thus, differentially expressed gene (DEG) and gene co-expression analyses are used to identify candidate genes (Rosati et al., 2024). In plants, triterpenoid biosynthesis usually occurs under biotic and abiotic stresses, and the genes related to triterpenoid synthesis get activated or upregulated due to increased triterpenoid accumulation after methyl jasmonate (MeJA) or dark treatment. Analyzing DEGs between treated and untreated plants has proven effective in identifying candidate genes related to triterpenoid synthesis. Han et al. utilized transcriptomic data from *C. asiatica* to screen 75 putative UGTs. Functional validation revealed that *Ca*UGT1 specifically transfers glucose to the C-28 carboxyl group of asiatic acid and madecassic acid (De Costa et al., 2017). Based on a similar strategy, Zhou et al. identified 51 UGTs from the *C. asiatica* transcriptome and found that *Ca*UGT73C7 and *Ca*UGT73C8 could catalyze asiaticoside synthesis while capturing *Ca*RRT that specifically transfer rhamnose. With these findings, the team achieved total synthesis of asiaticoside in *S. cerevisiae* with a yield of 772.3 μg/L (Zhao et al., 2024). The Sui team successfully identified two P450 enzymes and three UGTs as candidate genes for saikosaponin synthesis through the *B. chinense* transcriptome study (Sui et al., 2011).

The gene co-expression theory suggests that genes related to metabolic pathways have synergistic expression characteristics. Based on this principle, Chung et al. found a strong expression association between *Gm*CSyGT1 and soybean saponin synthesis genes in the *G. max* co-function network database (Chung et al., 2020). Using *Gm*yGTs as a probe, the researchers further identified *Gu*CSyGT and *Lj*CSyGT homologues in the transcriptome of *G. uralensis* and *L. japonicus*. Cross-species co-expression analysis confirmed the central role of CSyGTs in the synthesis of legume saponins, and the *S. cerevisiae* system verified that CsyGTs could catalyze the formation of glycyrrhetine-3-O-monoglucuronide (Chung et al., 2020). QS-21, a *β*-aromatic alcohol pentacyclic triterpene, contains three hydroxyl sites (glucuronic acid, galactose, xylose) and three carboxyl sites (fucose, rhamnose, xylose). The complexity of QS-21 molecular structure makes its chemical synthesis extremely challenging. Martin et al. analyzed the genes related to QS-21 synthesis based on the transcriptome data of saponins and achieved the compound heterologous synthesis in *tobacco* chassis through a 20-step catalytic reaction (Martin et al., 2024). Yao et al. performed RNA sequencing on different tissues of *A. elata (Miq.)*, screened 64 triterpene skeleton synthesis related genes, 254 CYP450 and 122 UGTs, and identified 5 oleanolic acid 3-O-glucosyl transferase candidate genes through expression profiling (Cheng et al., 2020). The research team also identified key UGTs gene clusters involved in triterpene glycosylation, including *Pg*UGT71A27, *Pv*fUGT1, *Pq*UGT1, etc., through transcriptome analysis of *P. ginseng*, *P. Vietnamensis*, *P. quinquefolius* and other species (Le et al., 2021; Sufang et al., 2024; Bihuan et al., 2024).

Transcriptome analysis offers a strategic approach for pinpointing UGT enzymes essential for triterpenoid modification. By tracking gene expression patterns under varying conditions, scientists can isolate environmentally responsive genes and validate their biochemical functions.

#### 2.1.3 Multiple omics screening of glycosyltransferases for triterpenoid glycosylation

Multi-omics integration strategy has shown unique advantages in gene function analysis (Luo, 2015). Integrating different omics data significantly improves the efficiency and accuracy of UGTs identification (Liu et al., 2022).

Integrated genomic and transcriptomic strategies effectively pinpoint UGTs involved in triterpene glycosylation. Advanced sequencing technologies create gene maps that identify functional genes and track their evolutionary changes (Wang and Huo, 2022). Transcriptome analysis tracks UGT expression patterns under various conditions, identifying key metabolic pathway elements (Zhang et al., 2025). Notable advances included Zhou et al. discovered of 42 saponin-related *Sm*UGTs distributed across 12 chromosomes in *S. mukorossi* (Zhou et al., 2024), and Wu et al. studied identifying 145 grapefruit UGTs through conserved motif analysis combined with developmental stage expression patterns (Wu et al., 2020). Jiang et al. analysed *P. notoginseng* data to isolate 27 UGTs, including *Pn*UGT33 that efficiently extended sugar chains on ginsenosides, achieving 51mg/L Rg3 yields in lab cultures (Jiang et al., 2022).

Combining transcriptomics and metabolomics to link gene activity to metabolite production enables precise functional analysis of UGT enzymes (He, 2022). This approach clarified how UGTs operate within biochemical pathways (He et al., 2021). Taking the non-model medicinal plant *A. flaccida* as an example, Zhan et al. systematically identified the enzymes involved in the triterpene saponins synthesis pathway, including the key enzymes of the mevalonate pathway (MVA) and the methylerythritol phosphate pathway (MEP), through combined transcriptome and proteome analysis. In addition, 126 CYP450 enzymes and 32 UGTs were identified as triterpene modification candidate genes (Zhan et al., 2016). Wang et al. used the combined transcriptomics and metabolomics to predict genes related to triterpene and flavonoid synthesis pathways in *B. rapa*, and found that ERF transcription factors may play an important role in the synergistic synthesis of the two kinds of compounds (Wang et al., 2024). Rai group has successfully discovered candidate genes for *C. officinalis* triterpene synthesis by combining LC-QTOF-MS metabolomics and RNA-seq transcriptome data (Rai et al., 2020). Through multi-omics analysis of different tissues of *B. chinense*, He et al. revealed the difference of saikosaponin synthesis pathways in roots, stems, leaves, and flowers, and found that the regulation of P450 genes Bc95697 and Bc35434 may improve saponin production (He et al., 2021). Chen et al. identified 69 terpenoids in *C. paliurus*, of which triterpenoids accounted for more than 80%, and identified key genes in the triterpenoid synthesis pathway by transcriptome co-expression analysis (Chen et al., 2024). These reseach demonstrated that multi-omics integration technology provides an effective case for exploring the mechanism of secondary metabolism in non-model plants.

Integrated genomic, transcriptomic, and metabolomic approaches have proven particularly effective for studying exploration of plant systems, from evolutionary relationships to specialized metabolite production (Fu et al., 2021; Yang et al., 2023). Liu et al. developed a multi-stage screening method that involved in triterpene saponin biosynthesis in *P. vulgaris*, using multi-omics integration to identify key biosynthetic genes. Their enzyme validation protocol established an efficient framework for the discovery of key enzymes including UGTs (Liu et al., 2025). Feng et al. mapped tissue-specific metabolic profiles in *I. hylonoma*, combining multi-omics data to mine regulatory genes controlling triterpene production, including CYP450 enzymes and transcription factors (Feng et al., 2024).

Liu et al. integrated multi-omics data from *P. vulgaris* using phylogenetic analysis and co-expression networks, successfully identifying two OSCs, three CYP716s, and four UGT73s from hundreds of gene family members. Heterologous expression confirmed the function of these genes matched computational predictions (Liu et al., 2025). Lin et al. identified four CYP genes, one UGT, and associated transcription factors as key regulators of triterpene saponin biosynthesis in *E. phaseoloides* through multi-omics analysis (Lin et al., 2022). *P. bretschneideri Rehd*. genome data, Li et al. predicted 178 UGTs and linked 11 to arbutin glycosylation using transcriptomic and metabolomic correlation studies, which promoted the understanding of the glycosylation mechanism in pear plants (Li et al., 2022). Martin et al. identified the triterpene glycosyltransferase UGT73CZ2 (Martin et al., 2024) through multi-omics analysis of *Q. saponaria*, and Hassan et al. found that CSLM1 and CSLM2 in *Q. saponaria* (Hassan et al., 2024), Liu et al. further analyzed UGT73CU3, UGT73CX2 and other series of transferases in *Q. saponaria* (Liu et al., 2024). Xu et al. targeted *Am*GT11 and *Am*GT36 based on the multi-omics data of *A. membranaceus* (Xu et al., 2024), and Feng et al. revealed the regulatory network of triterpene saponins synthesis through the multi-omics study of *I. hylonoma* (Feng et al., 2024). These cases demonstrated that multi-omics integration has become a powerful tool to analyze the complex metabolic networks in plants.

### 2.2 Screening of glycosyltransferases for triterpenoid glycosylation based on gene clusters

The structural complexity of natural products frequently mirrors the evolutionary sophistication of their biosynthetic gene clusters (BGCs) (Jensen, 2016). Molecular phylogenetics helps track gene evolution and identify relationships between related sequences. Notably, clustering patterns of terpenoid-modifying enzyme genes create opportunities for targeted gene discovery.

Specialized databases now exist for gene cluster prediction, including BAGEL4 (prokaryotic ribosome synthesis analysis), antiSMASH (bacterial/fungal/plant secondary metabolism), plantiSMASH (plant-specific cluster detection), and PRISM (secondary metabolism prediction) (Blin et al., 2023; Kautsar et al., 2018; Skinnider et al., 2020). For triterpene glycosyltransferase identification, plantiSMASH analysis of *Q. saponaria* genomes revealed candidate genes *Qs*0321930 and *Qs*0321920 near CYP716A297 (Guo et al., 2018), while later studies detected co-expression patterns between CSL genes and *Qs*bAS1 (Reed et al., 2023). Plant metabolic gene clusters have proven particularly valuable for research. The clustering tendency of terpenoid genes enables functional gene discovery through neighborhood analysis (Bharadwaj et al., 2021). In *A. chinensis* aescin synthesis, Sun et al. identified critical cluster components: *Ac*CYP716A275 (C-14 hydroxylation), *Ac*CYP716A278 (C-19 hydroxylation), *Ac*CSL1 (C-3 glucuronidation), and *Ac*BAHD3/6 (C-22 acetylation) (Sun et al., 2023b). These findings illuminate cluster component synergy and advance natural product biosynthesis understanding. Modern bioinformatics tools like BAGEL4 and antiSMASH now drive systematic BGC prediction and analysis (Jones et al., 2024; Chakraborty, 2022). These platforms accelerate both known gene validation and novel enzyme discovery, supporting metabolic pathway elucidation and pharmaceutical innovation.

### 2.3 Screening of Glycosyltransferases for triterpenoid glycosylation based on PSPG motif and phylogenetic analysis

**Figure 3:**
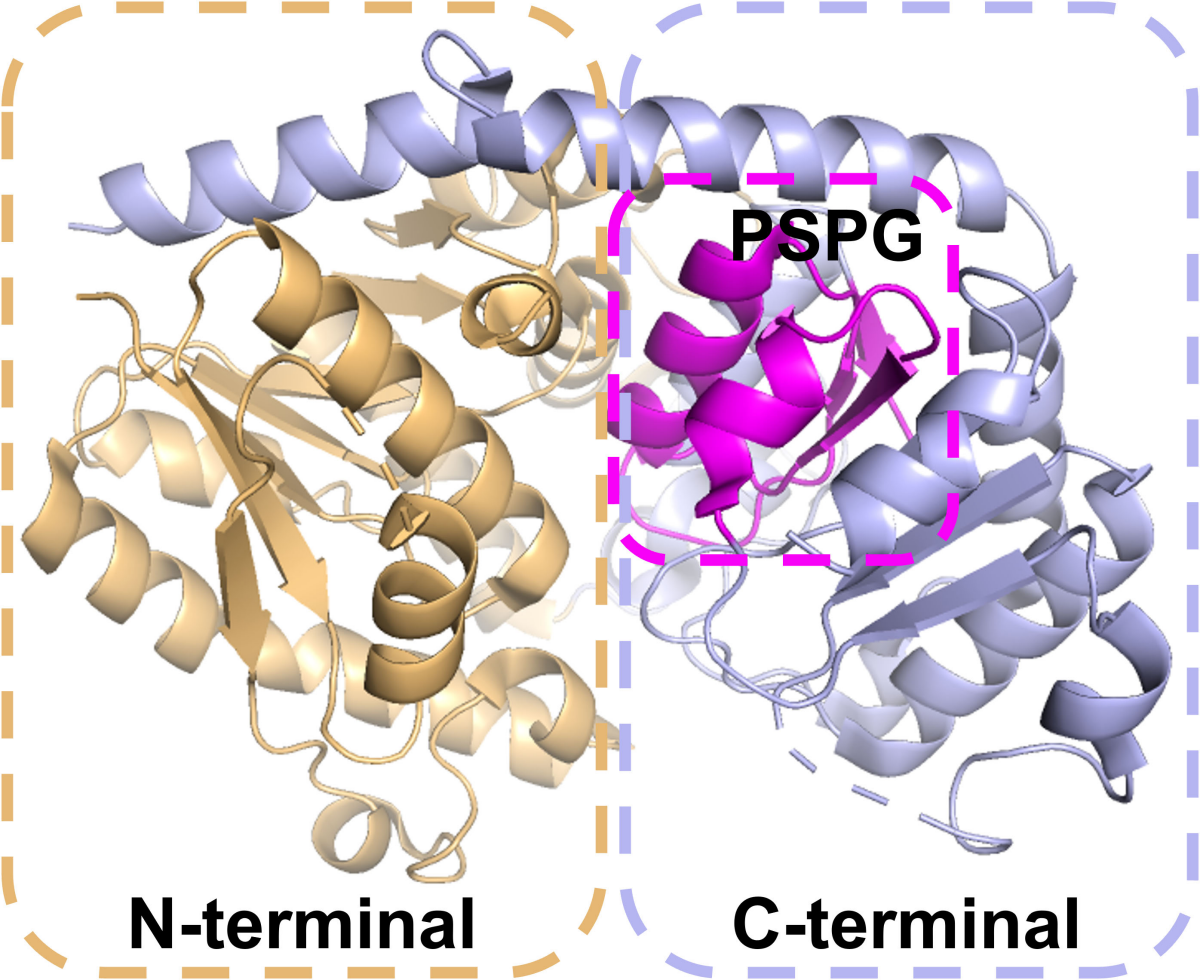
UGT 3D structure diagram.

The characteristic cleft between the N-terminal and C-terminal domains of UGTs forms a binding cavity that accommodates the donor and acceptor. The C-terminal conserved PSPG motif is the signature structural feature of UGTs (Wang et al., 2023a) (**Figure 3**), and some of its 44 amino acid residues are highly conserved, which is directly related to glycosyl donor selection (Chen et al., 2022). The histidine (H) at the terminal of PSPG motif determines galactose/arabinose transfer activity, whereas glutamine (Q) dominates glucose transfer properties. Based on this property, researchers remodeled the enzyme function by site-directed mutagenesis. The glucosyltransferase was obtained by replacing H404 of *As*AAT1 with proline (P154) by Louveau et al. (Louveau et al., 2018); Rahimi et al. converted the substrate specificity from UDP-Xly to UDP-Glc by the H to Q mutation of *At*UGT78D3 (Rahimi et al., 2019).

**Figure 4:**
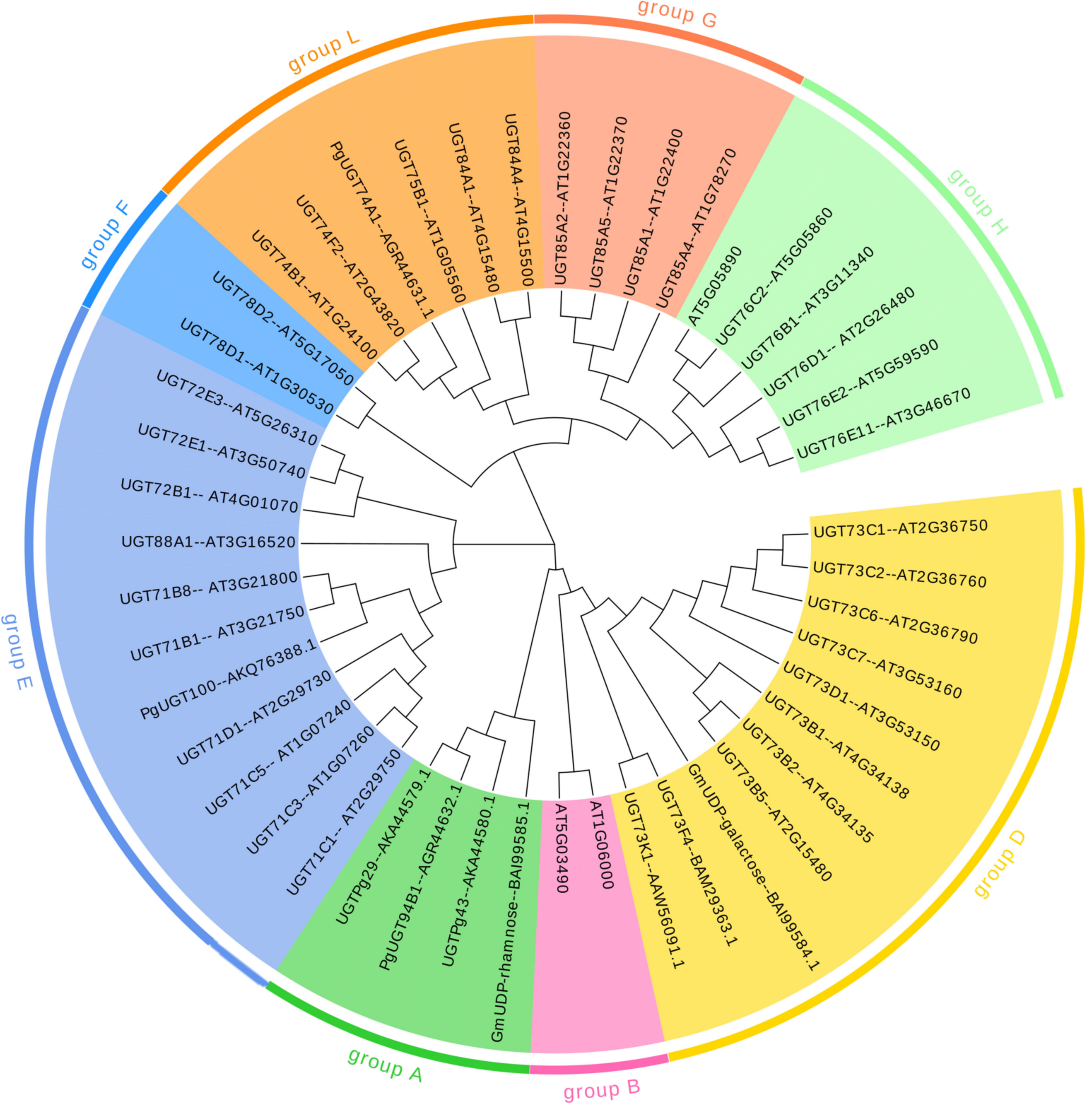
Phylogenetic tree analysis of UGTs. The tree is constructedbased on the PSPG motif sequences of UGTs involved in triterpenoidglycosylation. Key branches are annotated with PSPG groupings to highlight their substrate specificity.

**Figure 5:**
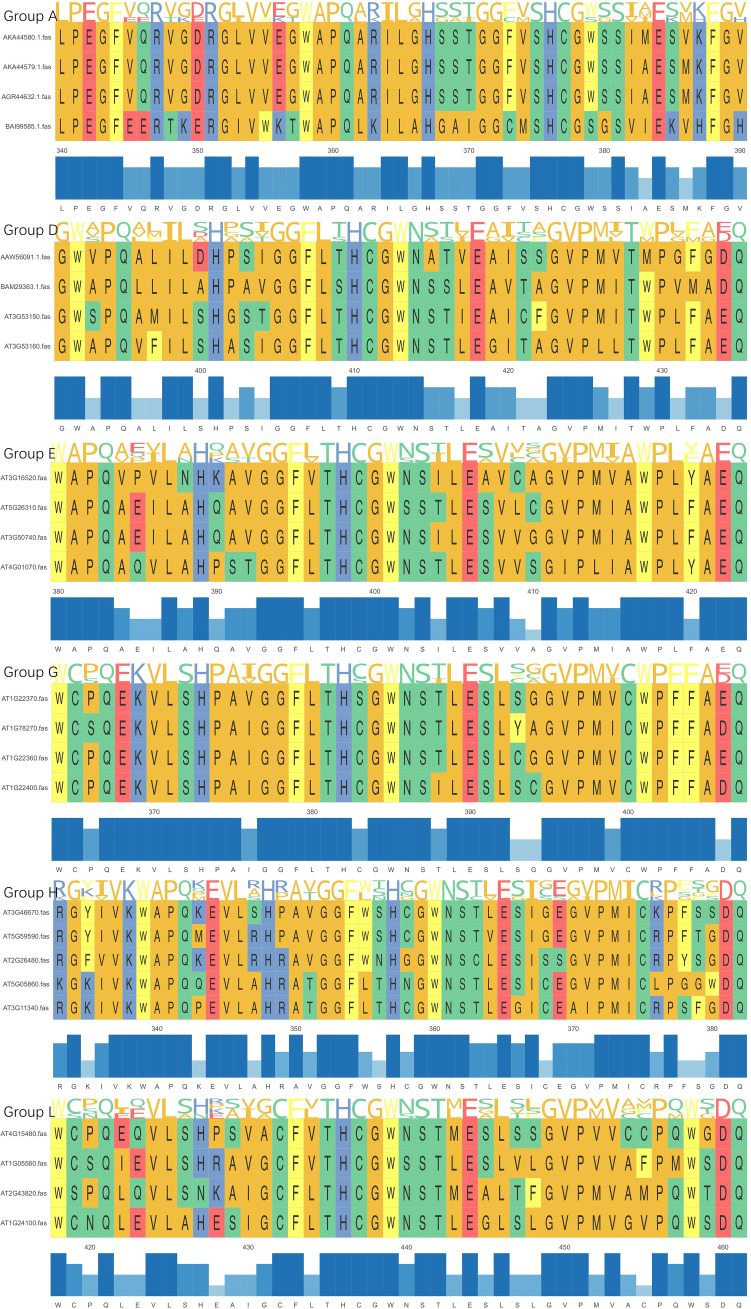
UGTs sequence alignment diagram.

Sequence variation in PSPG motifs can reflect functional differentiation. According to the N-terminal characteristic sequence, it can be divided into different subgroups: group A contains LPEGF, group D contains GW-PQ, group E contains WAPQ, group G contains WCPQ, group H contains RG-IV, and group L is characterized by WC-Q (Xueqing et al., 2022) (**Figures 4**, **5**). Novel gene substrate preferences can be predicted by comparing the known functions of Arabidopsis UGTs with triterpene UGTs (Rahimi et al., 2019). The phylogenetic analysis in this review revealed that UGTs with similar PSPG motifs presented clustering characteristics on the evolutionary tree (**Figure 4**), indicating their functional relevance. This combined analysis strategy based on phylogenetic trees and PSPG motifs provides a new idea for the functional prediction and targeted modification of UGTs.

### 2.4 Synthetic biology platform-based screening of glycosyltransferases for triterpenoid glycosylation

Wan et al. developed a modular yeast platform to dissect UGT-mediated glycosylation in plant natural products (PNPs), incorporating UDP-Glc and UDP-Xyl dependent enzymes (Wang et al., 2020). Their system employed a plug-and-play design to reconstitute PNP biosynthetic pathways in engineered yeast, enabling targeted investigation of glycosylation steps. Using this framework, they identified five triterpene glycosyltransferases from *P. notoginseng*, including the xylosyltransferase essential for the biosynthesis of Notoginsenoside R1.

Jian et al. elaborated the limited diversity of characterized UGT91H enzymes, a bottleneck in synthesizing trisaccharide-modified saponins. By analyzing evolutionary relationships among legume genomes which are rich in triterpenoid saponins, they selected 23 candidate genes using known UGT91H sequences as a seed. The results of functional identification confirmed conserved catalytic roles across 19 newly identified enzymes, demonstrating phylogenetic analysis as an efficient method for expanding this subfamily’s toolkit (Jian et al., 2024).

These parallel studies established complementary strategies: Wan et al. platform enables pathway reconstruction for functional validation, while Jian et al. phylogeny-driven approach accelerates enzyme discovery. Together, they advance both mechanistic understanding of plant glycosylation and practical tools for engineering bioactive compound production.

## 3 Catalytic mechanism of triterpenoid glycosyltransferases

**Figure 6:**
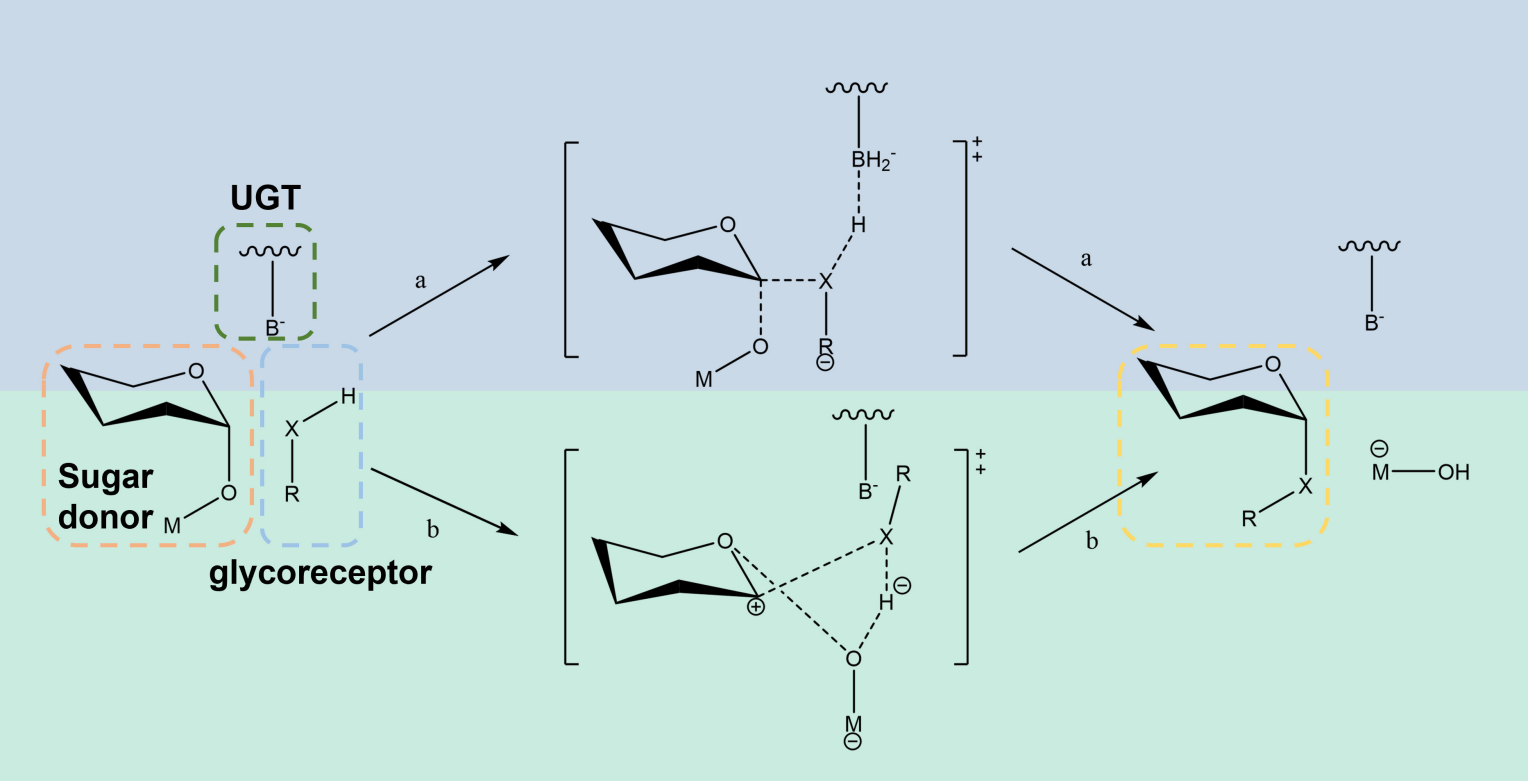
Catalytic mechanism of UGTs.

Triterpene saponins are consisted of glycosyl donors and a sugar acceptor, which are linked by glycosidylic linkages catalyzed by UGTs. Glycosylation can be classified into O-glycosidic (hydroxyl), C-glycosidic (carbon), N-glycosidic (amino) and S-glycosidic (sulfur) linkages according to the different linkage sites (Challinor and De Voss, 2013). Among them, O-glycosylation and C-glycosylation have attracted much attention because of their significant effects on the activity of compounds (Wang et al., 2023a). Glycosyltransferase catalysis generally follows the S_N_2 nucleophilic substitution mechanism (**Figure 6**), which consists of four key steps: substrate recognition, binding, glycosyltransfer, and product release. The active center of the enzyme binds to the substrate through hydrogen bonding, ion interaction and van der Waals force, thereby catalyzing the transfer of sugar groups from the donor to the acceptor (Xiao-chen et al., 2018).

The C-terminal domain of UGTs is primarily responsible for recognizing the UDP-glycosyldonor, whereas the N-terminus specifically binds receptor substrates (Osmani et al., 2009). Because the C-terminus of different UGTs needs to recognize the same or similar glycosyl donor, its conservation is higher than that of the N-terminal domain that recognizes heterologous receptors (Wang, 2009). An in-depth understanding of the catalytic mechanism of UGTs will not only help to reveal the law of natural product synthesis, but also provide a theoretical basis for enzyme engineering. Although natural UGTs play a central role in the synthesis of triterpene saponins, their catalytic efficiency, stability and substrate adaptability are often difficult to meet the needs of industrialization. Optimizing enzyme performance through protein engineering has become a current research hotspot.

### 3.1 Engineering of glycosyltransferases for enhanced catalytic properties

Synthetic biology constructs novel biosynthetic pathways through cross-species gene recombination. As the core components of the system, the natural catalytic properties of enzyme proteins often deviate from the industrial requirements, which restricts their wide application. Glycosylation modification of plant natural products is mainly mediated by UGTs (Siedhoff et al., 2020). However, the catalytic activity, stability, and substrate specificity of most natural UGTs are relatively low, which limits their application in the microbial biosynthesis of natural products. To address this challenge, enzyme modification technologies have been developed and can be broadly categorized based on their underlying principles: traditional enzyme engineering focuses on directed evolution, while rational and semi-rational redesign approaches leverage sequence and structural information to optimize natural enzymes (Guo et al., 2021) (**Figure 2c**).

#### 3.1.2 Homology modeling and molecular docking in protein research

Zhang et al. investigated the substrate recognition mechanism of UGT73F24 from *G. uralensis* towards glycyrrhetinic acid and UDP-glucose using homology modeling and molecular docking analysis. Based on the identified recognition mechanism, they selected amino acids located near the C3-OH group of glycyrrhetinic acid and the glucose moiety of UDP-glucose, as well as residues in the substrate-binding pocket, as candidate sites for site-directed mutagenesis. They identified two key residues, I23 and L84, and the combination of mutations I23G/L84N resulted in a 4.1-fold increase in activity (Zhang et al., 2020). In a similar study, Chen et al. analyzed the hydrogen bond interactions between UGT76G1 from *S. rebaudiana* and UDP-glucose. They identified the conserved residues His17 and Asp359, and selected Asn358, located near the substrate channel, as a target for saturation mutagenesis. The N358F mutant, when incorporated into a multi-enzyme reaction system, led to a 60% increase in the yield of rebaudioside D (Chen et al., 2020). Bi et al. engineered the 1,6-glucosyltransferase *Ca*UGT3 by substituting valine for residue T145, thereby converting the enzyme into a xylosyltransferase capable of catalyzing the conversion of cinnamyl alcohol monoglucoside (rosin) into rosavin E. Further enhancement of enzyme activity (2.9-fold increase) was achieved by introducing the N375Q mutation. The synthesis of rosavin E from glucose was successfully carried out, reaching a final concentration of 92.9 mg/L, by combining the *Ca*UGT3T145V/N375Q variant with UDP-Xly synthase from *S. meliloti* 1021 (*Sm*UXS) and the enzymes involved in rosin biosynthesis, all expressed in a phenylalanine-overproducing *E.coli* strain (Bi et al., 2022). Li et al. aimed to address the relatively low catalytic efficiency of triterpene-class UGTs. They selected UGT74AC1, a glycosyltransferase from *S. grosvenorii*, and analyzed its crystal structure. Using this structural insight as a foundation, they applied directed evolution and sequence/structure-based engineering to enhance its catalytic properties. Several resulting UGT variants demonstrated a remarkable 10^2^- to 10^4^-fold improvement in catalytic efficiency for triterpene glycosylation. One of these mutants has 4.17 × 10^4^ times higher catalytic efficiency against mogrol and 1.53 × 10^4^ times higher catalytic activity against UDP-glucose (Li et al., 2020). Chen et al. identified the *Am*GT1G146V/I mutants through sequence alignment, molecular docking, and site-directed mutagenesis, which specifically utilize UDP-Xyl while showing no activity toward UDP-Glc. By combining *Am*GT1/5/9 and *Am*GT1G146V/S with the previously reported *Am*GT8 and its mutant *Am*GT8A394F, they accomplished the combinatorial synthesis of 13 cycloartane-type saponins from *A. membranaceus* (Chen et al., 2023).

#### 3.1.2 Directed evolution of enzymes for improved catalytic performance

**Figure 7:**
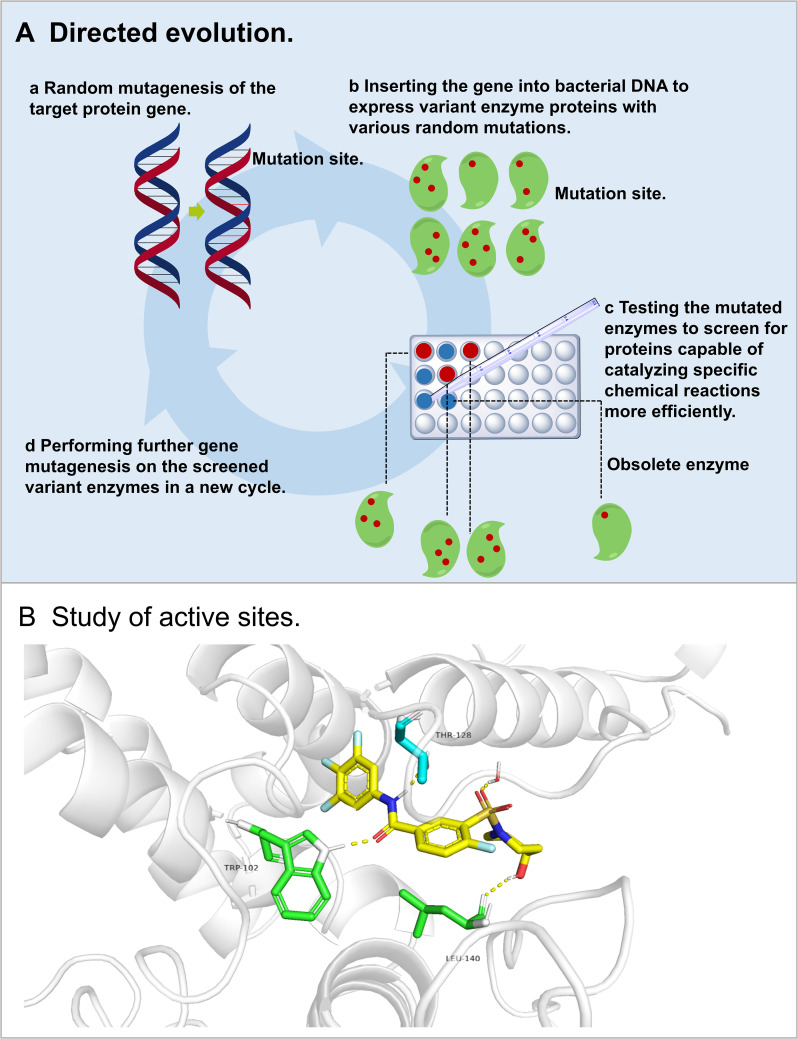
Directed evolution. **(A)** process of directed evolution; **(B)** study of active sites.

Directed evolution mimics natural gene mutation and selection processes through repeated mutagenesis and screening (Sinha and Shukla, 2019). This approach identifies enzyme variants with increased activity, substrate specificity, or thermal stability using methods, such as random mutagenesis, site-directed mutagenesis, and DNA recombination (**Figure 7A**). Although powerful for engineering triterpene-producing enzymes, the technique faces challenges in managing vast mutation spaces during combinatorial mutagenesis, complicating optimization efforts (Thieker et al., 2022).

As for the biosynthesis of ginsenoside, low catalytic efficiency of UGTs often limits the yields. Researchers solved it by creating mutant libraries via error-prone PCR and screening variants through HPLC or fluorescent assays. This strategy was applied to *Pg*UGT74AE2, resulting in mutants with higher catalytic efficiency and improved ginsenoside production (Ji et al., 2025). To expand the substrate range, glycyrrhizin pathway studies combined DNA recombination with rational design on *Gm*UGAT, and engineered mutants capable of processing non-native substrates (Guo et al., 2021). Thermal stability improvements were achieved by evolving UGT variants through combinatorial mutagenesis, yielding heat-tolerant enzymes for industrial production (Bian et al., 2024).

The above cases demonstrated the capacity of directed evolution to increase enzyme activity by screening random mutations for desirable traits. The improved catalytic efficiency of *Pg*UGT74AE2 reflected the improved yield, while the *Gm*UGAT modification expanded the substrate range through optimization of the binding pocket. Despite the challenges in navigating extensive mutation field, technological advances promise to streamline mutant screening processes.

#### 3.1.3 Rational and semi-rational design in enzyme engineering

Rational and semi-rational design have become fundamental approachfor enhancing enzymatic capabilities through structural and functional insights. These approaches commonly employ targeted amino acid substitutions or combinatorial mutation libraries, focusing on improving catalytic traits. Existing sequence databases combined with crystallographic evidence allow accurate identification of mutation hotspots, substantially streamlining experimental workflows while maintaining discovery efficiency. These strategies also illuminate fundamental structure-function relationships underlying enzymatic enhancement. Active site residues and adjacent regions are typically grouped into distinct layers based on their distance from the binding substrates. Structural alterations within active site residues occasionally yield unexpected stereochemical refinements, whereas mutations at sites farther from the active center often lead to measurable improvements in thermal tolerance and operational stability (Patsch et al., 2024) (**Figure 7B**). The expanding repository of high-resolution protein structures, augmented by computational modeling advancements, has enabled new frameworks for systematic enzyme engineering. Contemporary semi-rational platforms like 3DM and HotSpot Wizard integrate multidimensional structural data with evolutionary information, demonstrating particular promise for tailoring biocatalyst performance (Cho et al., 2021; Bendl et al., 2016). For instance, 3DM uses structure-based multiple sequence alignments to identify key residues affecting enzyme activity, drawing on structural-functional relationships from databases like PDB, GenBank, PubMed, and Swiss-Prot. These predictions are subsequently validated through multi-site saturation mutagenesis. Similarly, HotSpot Wizard integrates multiple databases and computational tools, both structural and evolutionary, to predict residues that influence enzyme stability, activity, and substrate specificity.

The practical application of these methods is evident in various case studies. Zhang et al. investigated *A. membranaceus* and discovered the first cycloartane-type triterpene glycosyltransferase, *Am*GT8, which catalyzes two consecutive glycosylation reactions at the 3-OH and 2-OH positions of cycloastragenol. Through targeted semi-rational engineering, researchers developed three *Am*GT8 variants (A394D, A394F, T131V) that showed specialized glycosylation patterns at distinct oxygen positions. These engineered catalysts enabled efficient biosynthesis of Astragalus-derived saponins through accurate glycosyl moiety attachment (Zhang et al., 2022). In parallel work, Xie et al. used a structurally directed mutagenesis technique to redesign the 4*α*GT from Synechocystis sp. PCC 6803. By analyzing residue spatial configurations through HotSpot Wizard, they created iincreased variants that improved enzymatic hydrolysis efficiency, subsequently scaled for commercial production (Jingwen et al., 2024). Comparative analysis of UGT91H_A1 and UGT91H8 revealed an important RTAS motif (R212/T213/A214/S215) governing substrate preference. Transplantation of this sequence into UGT91H8 resulted in the UGT91H8_6mu variant, achieving 15.9% catalytic conversion for compound 17 through optimized binding geometry. Computational modeling showed how this motif regulates substrate-channel interactions (Jian et al., 2024). Further reseach demonstrated that *Gu*GT14 engineering yielded H47P and I182L mutants exhibiting 101.74% and 405.78% activity respectively versus wild-type. These optimized catalysts significantly improved Rh2 ginsenoside yields, establishing viable production routes for this pharmaceutically valuable compound (Yan et al., 2024).

Effective enzyme engineering requires a detailed understanding of structural-functional relationships governing catalytic behavior, which enables accurate alterations at key amino acid positions to achieve desired enzymatic improvements (Qian et al., 2021). The combination of rational and semi-rational design approaches has proved very effective in enzyme engineering, not only providing insights into the mechanisms of enzyme function.

## 4 Synthesis of glycosyl donors for glycosylation reactions

**Figure 8:**
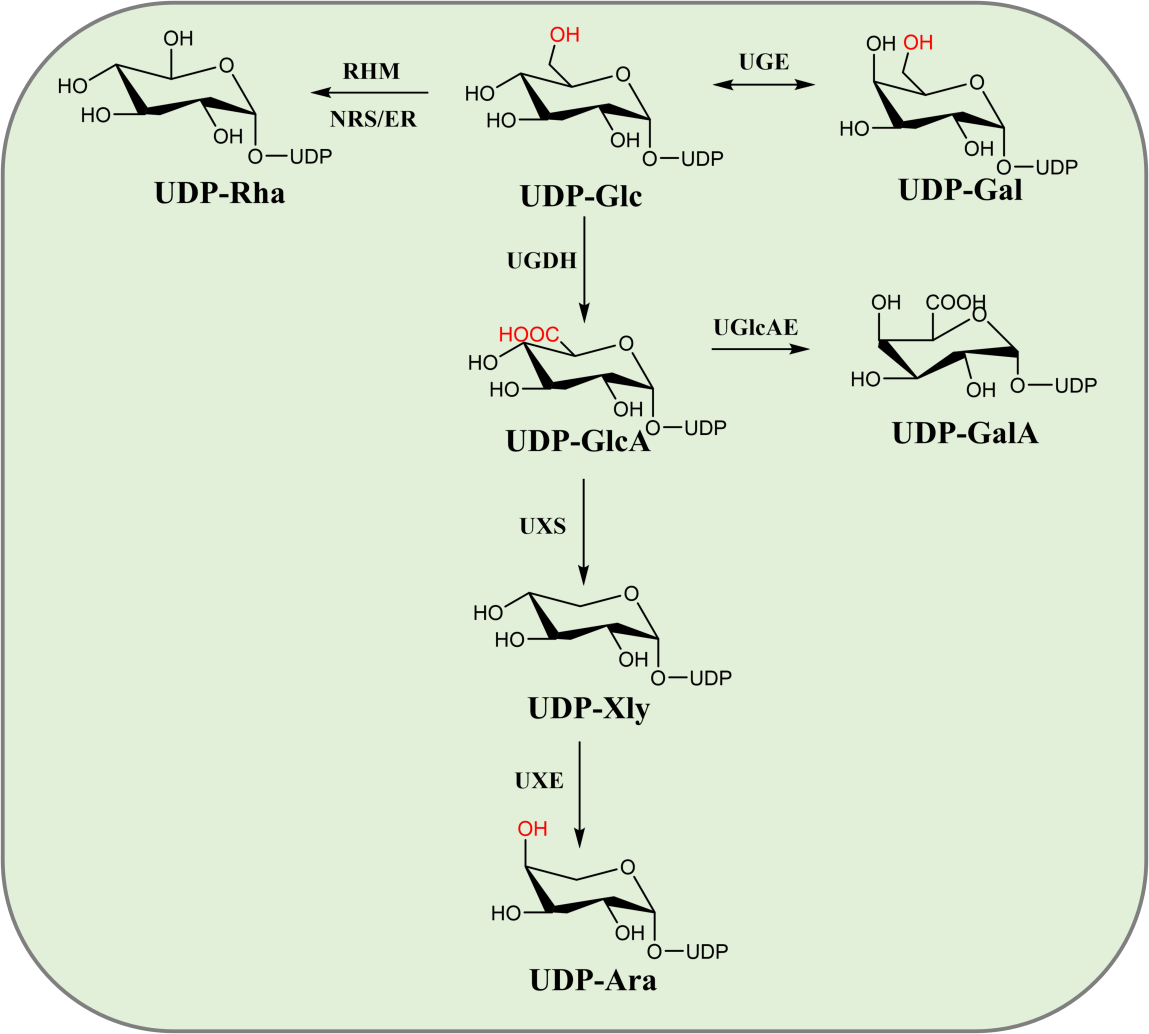
Glycosyl donor interconversion diagram.

Triterpenoid saponins are composed of a glycoside moiety and an aglycone linked by glycosidic bonds, catalyzed by UGTs. The substrates for these enzymes include glycosyl acceptors and donors. However, the diversity of glycosyl donors in most host cells is limited, which may not meet the demands for synthesizing complex natural compounds. Various glycosyl donors can be interconverted (**Figure 2d**). Sucrose, under the catalysis of sucrose synthase (SUS), can produce UDP-Glc. The conversion of UDP-Glc to UDP-Ara involves three key metabolic enzymes in a three-step process: dehydrogenation, decarboxylation and epimerization. UDP-Glc is converted to UDP-GlcA by UDP-glucose dehydrogenase (UGDH). UDP-GlcA is then catalyzed by UDP-xylose synthase to form UDP-Xyl. UDP-Xyl is enzymatically transformed into UDP-Ara through UDP-xylose-4-epimerase (UXE)-mediated structural rearrangement. Meanwhile, rhamnose mutase (RHM) catalyzes the conversion of UDP-Glc to UDP-Rha, while UDP-arabinose 4-epimerase (UAXS) processes UDP-GlcA into UDP-Cel. The same UDP-GlcA precursor can alternatively yield UDP-galacturonic acid via UGlcAE activity (Sen and Jian-qiang, 2016) (**Figure 8**). These biochemical pathways have driven significant interest in synthesizing diverse glycosyl donors through exogenous gene integration, both in laboratory settings and biological systems.

### 4.1 *In Vitro* synthesis of glycosyl donors for glycosylation reactions

**Figure 9:**
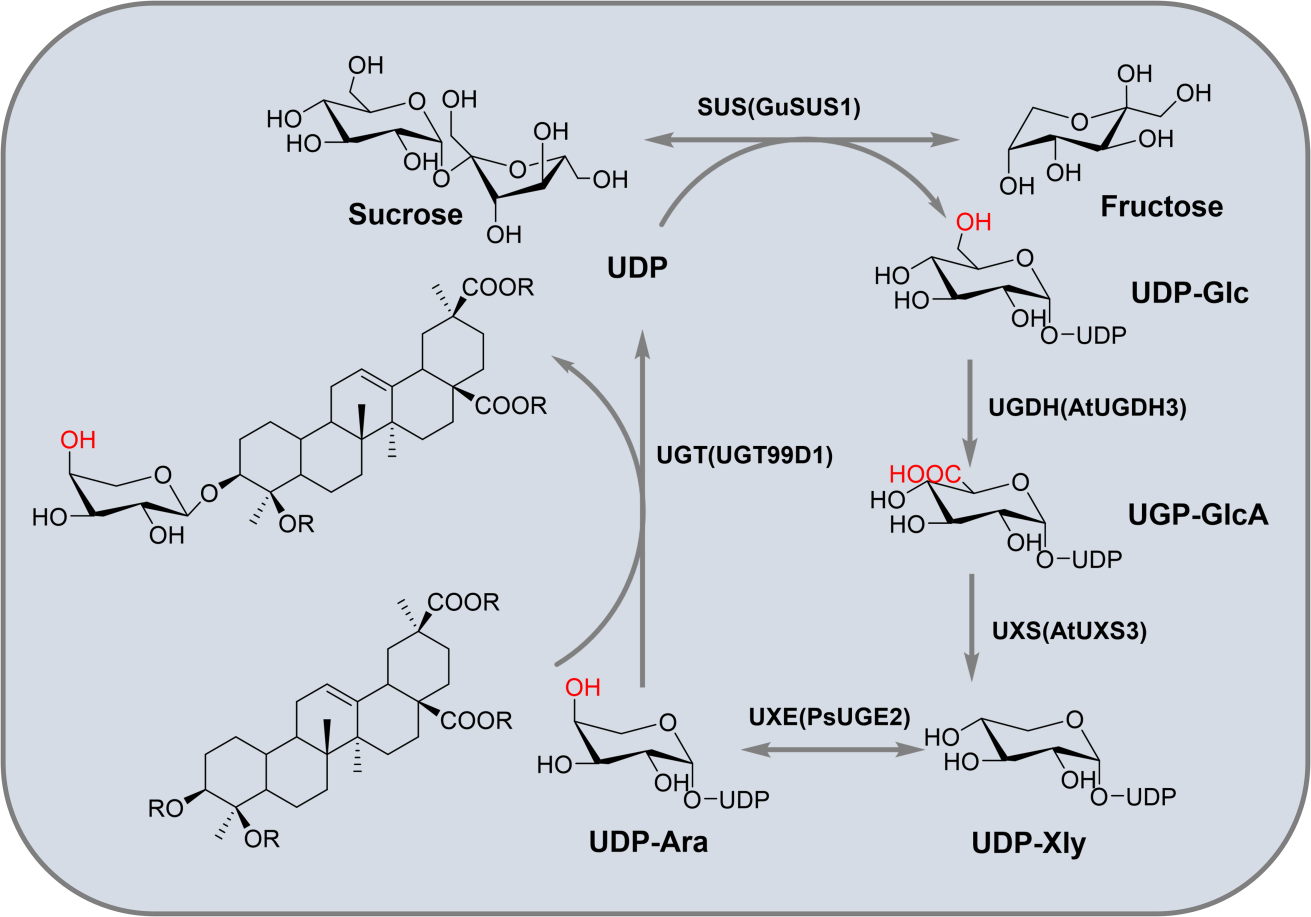
Synthetic Ara-BA *in vitro*.

*In vitro* synthesis of glycosyl donors offers flexibility and cost-effectiveness. Sun et al. screened several key enzymes to facilitate the production of glycosyl donors in microbial systems. These included *Gu*SUS1 from *G. uralensis*, *At*UGDH3 and *At*UXS3 from *A. thaliana*, and *Ps*UGE2 from *P. sativum*, which collectively enable the conversion of sucrose into arabinose. The successful production of arabinose was confirmed by using the arabinose transferase UGT99D1 in *A. sativaL* (Sun et al., 2023a). This strategy not only facilitates the generation of glucose, glucuronic acid, xylose, and arabinose donors, but also addresses a significant challenge in the biosynthesis of triterpenoid saponins. Lay the foundation for the microbial synthesis of natural glycoside compounds. They successfully achieved the *in vitro* production of UDP-Ara and catalyzed the synthesis of Ara-BA *in vitro* (**Figure 9**).

UDP-glucose transferase can be coupled with sucrose synthase to construct synthetic pathways with different glycosyl donors, using inexpensive sucrose as the substrate for the glycosylation of natural products. However, the hydrolysis of sucrose leads to the accumulation of by-product fructose, which reduces the atomic economy of sucrose and inhibits *in situ* UDP recycling. To improve sucrose utilization, Wang et al. first demonstrated that a polyphosphate-dependent glucose kinase can convert fructose into fructose-6-phosphate without relying on expensive ATP. They then introduced the glucose kinase into the UDP-2E recycling system, constructing an improved three-enzyme UDP (UDP-3E) recycling system. This system enhances the glycosylation efficiency of triterpenoids by phosphorylating fructose, accelerates sucrose hydrolysis and UDP recycling, and improves the overall utilization of sucrose (Wang et al., 2023b).

Song et al. conducted a genomic analysis of the Pacific oyster and identified three genes responsible for catalyzing the conversion of UDP-Gal, UDP-GlcA, and UDP-Xyl. Enzymatic assays demonstrated that CGIUGE catalyzes the conversion of UDP-Glc to UDP-Gal, CGIUGD catalyzes the conversion of UDP-Glc to UDP-GlcA, and CGIUXS catalyzes the conversion of UDP-GlcA to UDP-Xyl. These findings enable the *in vitro* synthesis of UDP-Gal, UDP-GlcA, and UDP-Xyl using UDP-Glc as the starting substrate (Song, 2017).

Yin et al. cloned 17 key enzyme genes (*Oc*UXS1-6, *Oc*UAXS1/2, *Oc*UGE1/2, *Oc*UXE1/2, *Oc*RHM1, *Oc*NER1, *Oc*GlcAE1/2/3) involved in the biosynthesis of diversified natural small molecule glycosides from *O. caudatum*, which catalyze the formation of key sugar nucleotide donors such as UDP-Xyl, UDP-Ara, UDP-Gal, UDP-GalA, and UDP-Rha. Through functional characterization of these enzymes, they successfully achieved the *in vitro* synthesis of UDP-Xyl, UDP-Ara, UDP-Gal, UDP-GalA, and UDP-Rha using UDP-Glc as a precursor (Yin, 2016). Jian et al. achieved *de novo* synthesis of UDP-Rha *in vitro* by coupling the *Gu*SUS1-Δ9, *At*RHM2, and *At*NRS/ER genes. The UDP-Rha regeneration system, combined with UGT, holds great potential for the efficient production of glycosylated triterpenoid compounds (Jian et al., 2024). These studies suggest that *in vitro* synthesis holds great potential for the production of diverse glycosyl donors. They also highlight the potential of *E. coli* as a host for glycosyl donor synthesis.

### 4.2 The synthesis of glycosyl donors within microorganisms

The limitations of *in vitro* glycosyl donor synthesis highlight the advantages of microbial production systems that are more efficient, cost-effective, and scalable. Three microbial platforms including *E. coli*, *S. cerevisiae*, and *B. subtilis* dominate current research. The robust metabolism and rapid growth of *E. coli* enable efficient protein production, with shorter cultivation cycles making it ideal for industrial-scale operations (Rosano et al., 2019). *S. cerevisiae* stands out for its eukaryotic machinery, particularly the endoplasmic reticulum, which supports plant enzyme function, and a natural UDP-Glc reserve that can facilitate synthesis of glycosyl donor precursors (Long et al., 2024). *B. subtilis* is prominent in recombinant protein secretion and application in agricultural biotechnology (Pramastya et al., 2021).

Li et al. achieved *de novo* synthesis of UDP-Ara through coordinated expression of UGDH, XylS, and UXE genes, resulting in rosavin production. Bi et al. optimized UDP-Xly synthesis by overexpressing *At*UXS3/*Sm*UXS to produce rosavin E (Bi et al., 2022). Liu et al. established UDP-Rha/Gal biosynthesis pathway in *E. coli* to produce six different flavonoid glycosides (Liu et al., 2023). Zhao et al. demonstrated the complete madecassoside synthesis through VvRHM-NRS-mediated UDP-Rha conversion (Zhao et al., 2024).

*S. cerevisiae* platforms utilizes native UDP-Glc pools for donor synthesis. Oka and Jigami engineered yeast strains expressing plant *At*UGD1/*At*UXS3 to convert UDP-Glc to UDP-Xyl (Oka and Jigami, 2006). Zhang et al. achieved 2.53 g/L 2’-fucosyllactose production through integrated lactose/GDP-Fuc modules (Zhang et al., 2024b).

These microbial engineering strategies address critical challenges in triterpenoid saponin biosynthesis by diversifying glycosyl donor availability (Guihang et al., 2023). While each platform demonstrates unique strengths –the scalability of *E. coli*, the eukaryotic compatibility of yeast, and the secretion capacity of *B. subtilis*– future progress requires optimized chassis engineering (Xu et al., 2020; Shi and Jiang, 2020) and novel host exploration (Tianli et al., 2024). Strategic integration of synthetic biology tools will drive industrial-scale production of bioactive glycosides (**Table 2**).

**Table 2:** Advantages and disadvantages of glycosyl donors synthesis.

Strategies for glycosyl donors synthesis	Advantage	Disadvantage
*In vitro* synthesis	Highly controllable. High product purity. Strong flexibility.	High cost. Complex reaction steps. Difficult to scale up, more suitable for laboratory research.
Synthetic in *E. coli*	Low cost and easy to culture. Simple genetic manipulation. Strong scalability, suitable for industrial production. Environmentally friendly.	Metabolic balance is challenging. Substrate utilization limitations, unable to efficiently utilize certain carbon sources (e.g., sucrose). Glycosyl donors purity issues, with numerous by-products. Gene expression stability issues. Metabolic bottlenecks, such as insufficient UDP-Glc supply. Product purity concerns.
Synthesized in *S. cerevisiae*	Efficient sugar metabolism. Clear biosynthetic pathway. Yeast-based synthesis of glycosyl donors is more environmentally friendly compared to chemical synthesis. Strong scalability, suitable for industrial production.	
Synthesized in *B. subtilis*	High safety. Strong metabolic engineering capabilities. Efficient whole-cell catalytic activity. Strong scalability, suitable for industrial production. Environmentally friendly.	

## 5 Conclusion and future perspectives

In recent years, triterpene saponins have made important breakthroughs in the discovery and modification of UGTs and the synthesis of glycosyl donors (Li et al., 2023). These advances not only deepen the understanding of the biosynthetic mechanism of triterpene saponins, but also open up a new path for their industrial production. Using microbial platforms to synthesize triterpenoids has become an efficient and sustainable biomanufacturing strategy: Liu et al. achieved full synthesis of a complex vaccine adjuvant QS-21 in engineered yeast in 2024 (Liu et al., 2024); In the same year, Martin et al. completed the whole biosynthesis of QS-21 in *tobacco* by integrating a 20-step reaction (Martin et al., 2024). The Zhao team first synthesized asiaticoside in *S. cerevisiae* using oxidase and glycosyltransferase, and the yield reached 772.3 μg/L (Zhao et al., 2024). Wang et al. increased ginsenoside Rh2 production to 2.25 g/L by fed-feed fermentation (Wang et al., 2019). Microbial *de novo* synthesis of triterpenoid compounds such as ginsenosides has been achieved by many teams around the world through systematic metabolic engineering strategies such as heterologous pathway reconstruction and precursor supply optimization (Ren et al., 2022). The core of such platforms lies in pathway design, dynamic regulation and cross-scale optimization. With the development of synthetic biology tools such as CRISPR and AI enzyme design, the industrial production of more high-value triterpenoid compounds is expected to gradually replace the traditional plant extraction method (Li et al., 2023; Kim et al., 2017) (**Figure 2e**).

In terms of UGTs discovery and identification, the combination of genome-transcriptome-metabolome studies has significantly improved the UGTs screening efficiency (Chen et al., 2024). Gene cluster mapping, phylogenetic analysis, and application of PSPG motifs provide new insights into UGTs function (Zhan et al., 2022; Gharabli and Welner, 2024). In UGTs engineering, rational and semi-rational design strategies have effectively improved enzyme activity, substrate specificity, and stability (Chica et al., 2005). By using directed evolution, UGTs mutants with 2–5 times higher catalytic efficiency were obtained (Zhang et al., 2024a). In the field of glycan donor synthesis, the strategy of exogenous gene introduction has successfully overcome the limitation of the diversity of glycan donors in host cells: engineered microorganisms such as *E. coli* and yeast have been able to efficiently synthesize a variety of glycan nucleotides (Li et al., 2024; Oka and Jigami, 2006).

Despite progress, the field still faces many challenges. Functional annotation of UGTs requires a large number of experimental verification and the complexity of gene clusters and plant genome diversity increase the difficulty of comprehensive identification. The prediction of mutation effects in UGTs engineering is still challenging, and directed evolution is limited by the large mutation space and high-throughput screening requirements. The cost of *in vitro* synthesis of glycosyl donors is high, and the *in vivo* synthesis needs to optimize the metabolic network to improve the efficiency (Zhou et al., 2022).

In the future, it is necessary to deeply integrate multi-omics data to analyze the triterpene synthesis regulatory network (Chai et al., 2023) and develop a high-throughput screening platform to accelerate UGTs validation and glycodonor pathway optimization (Kwon et al., 2024). The application of machine learning and AI technology in enzyme function prediction and metabolic pathway design will accelerate the construction of efficient microbial factories (Jang et al., 2022). Developing a novel microbial host that can expand the synthesis pathway of glycans donors (Tianli et al., 2024) and in-depth understanding of the regulatory mechanism of triterpene synthesis will help to improve the yield of engineered strains (Yuanyuan et al., 2021). Continuous technological innovation will promote the industrial application of triterpene saponins in medicine, food and other fields.
